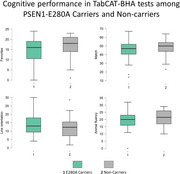# Digital Cognitive Markers to Characterize Preclinical Stages of autosomal‐dominant Alzheimer's Disease: A Preliminary Study

**DOI:** 10.1002/alz70856_105619

**Published:** 2026-01-09

**Authors:** Stella Maris Valencia, Juan Pablo Sanchez, David Fernando Aguillón Niño, Ana Y Baena, Natalia Londono, Susana Ramirez, Sandra P. Trujillo, Diana Gomez, Diego Camilo Díaz, Katherine L. Possin, Natalia Trujillo Orrego

**Affiliations:** ^1^ Grupo de investigación en salud mental, Universidad de Antioquia, Medellin, Antioquia, Colombia; ^2^ Grupo de Neurociencias de Antioquia, Facultad de Medicina, Universidad de Antioquia, Medellín, Antioquia, Colombia; ^3^ University of Antioquia, Medellín, Antioquia, Colombia; ^4^ Grupo de Neurociencias de Antioquia, Facultad de Medicina, Universidad de Antioquia, Medellín, Colombia; ^5^ Fundación Universitaria Luis Amigó, Medellín, Antioquia, Colombia; ^6^ Memory and Aging Center, University of California San Francisco, San Francisco, CA, USA; ^7^ Global Brain Health Institute (GBHI), University of California San Francisco (UCSF); & Trinity College Dublin, San Francisco, CA, USA; ^8^ Atlantic Fellow for Equity in Brain Health at University of California, San Francisco / Trinity College of Dublin, San Francisco, CA, USA; ^9^ University of Antioquia, Medellin, Colombia; ^10^ Florida International University, Miami, FL, USA

## Abstract

**Background:**

Alzheimer's disease and related dementias are rising at an alarming rate (>300%), leading to human and economic losses, particularly in resource‐limited low‐ and middle‐income countries. This exacerbates health disparities and underscores the urgent need for scalable early detection tools to support preventive intervention strategies. Emerging digital cognitive assessment tools, such as TabCAT‐BHA, a 15‐minute tablet‐based battery, offer a practical alternative for identifying early cognitive markers. Colombia's autosomal dominant PSEN1‐E280A genetic variant cohort, causative of early‐onset familial AD, provides a unique opportunity to examine preclinical cognitive changes associated with the disease. As the need for innovative cognitive markers rises, evidence on their validity is essential for determining their clinical utility. The objective is to evaluate the validity of TabCAT‐BHA for detecting cognitive differences between asymptomatic PSEN1‐E280A carriers and non‐carriers and correlating with established, traditional cognitive tests of the same domains.

**Method:**

This cross‐sectional study included 135 asymptomatic participants: 79 PSEN1‐E280A carriers and 56 non‐carriers, mean age: 32.5 years. TabCAT‐BHA encompasses memory, executive function, processing speed, visuospatial ability, and language cognitive measures. Neuropsychological paper‐pencil assessment was carried out for similar domains through a 25‐minute evaluation using widely used tests. Descriptive statistics summarized demographic and cognitive performance. Independent sample t‐tests were used to assess cognitive performance differences between groups. Correlation coefficients evaluated concurrent validity between TabCAT‐BHA and traditional paper‐pencil neuropsychological tests.

**Results:**

Both groups were comparable in terms of sex, age, and educational level. Non‐carriers demonstrated a trend toward better performance across all cognitive domains. Statistically significant differences were observed in TabCAT‐BHA's memory, visuospatial ability, and language metrics between groups, while traditional evaluations identified differences only in memory tests. Correlation analyses revealed moderate relationships between TabCAT‐BHA and traditional tests across same domains, with lower correlations with different domains.

**Conclusion:**

TabCAT‐BHA detected broader cognitive differences across multiple domains between PSEN1‐E280A mutation carriers and non‐carriers, underscoring its utility in identifying early cognitive changes during the preclinical stage of AD. Its demonstrated concurrent validity with traditional evaluations further highlights its potential as a scalable, efficient tool for preclinical AD detection, particularly in low‐ and middle‐income countries where resource constraints limit access to traditional diagnostic approaches.